# Identification of clinical factors related to prediction of alcohol use disorder from electronic health records using feature selection methods

**DOI:** 10.1186/s12911-022-02051-w

**Published:** 2022-11-23

**Authors:** Ali Ebrahimi, Uffe Kock Wiil, Amin Naemi, Marjan Mansourvar, Kjeld Andersen, Anette Søgaard Nielsen

**Affiliations:** 1 SDU Health Informatics and Technology, The Maersk Mc-Kinney Moller Institute, University of Southern Denmark, Odense, Denmark; 2 Department of Mathematics and Computer Science, University of Southern Denmark, Odense, Denmark; 3 Unit for Clinical Alcohol Research, Clinical Institute, University of Southern Denmark, Odense, Denmark

**Keywords:** Alcohol use disorder, Clinical factor identification, Gender disparity, Machine learning, Feature selection

## Abstract

**Background:**

High dimensionality in electronic health records (EHR) causes a significant computational problem for any systematic search for predictive, diagnostic, or prognostic patterns. Feature selection (FS) methods have been indicated to be effective in feature reduction as well as in identifying risk factors related to prediction of clinical disorders. This paper examines the prediction of patients with alcohol use disorder (AUD) using machine learning (ML) and attempts to identify risk factors related to the diagnosis of AUD.

**Methods:**

A FS framework consisting of two operational levels, base selectors and ensemble selectors. The first level consists of five FS methods: three filter methods, one wrapper method, and one embedded method. Base selector outputs are aggregated to develop four ensemble FS methods. The outputs of FS method were then fed into three ML algorithms: support vector machine (SVM), K-nearest neighbor (KNN), and random forest (RF) to compare and identify the best feature subset for the prediction of AUD from EHRs.

**Results:**

In terms of feature reduction, the embedded FS method could significantly reduce the number of features from 361 to 131. In terms of classification performance, RF based on 272 features selected by our proposed ensemble method (Union FS) with the highest accuracy in predicting patients with AUD, 96%, outperformed all other models in terms of AUROC, AUPRC, Precision, Recall, and F1-Score. Considering the limitations of embedded and wrapper methods, the best overall performance was achieved by our proposed Union Filter FS, which reduced the number of features to 223 and improved Precision, Recall, and F1-Score in RF from 0.77, 0.65, and 0.71 to 0.87, 0.81, and 0.84, respectively. Our findings indicate that, besides gender, age, and length of stay at the hospital, diagnosis related to digestive organs, bones, muscles and connective tissue, and the nervous systems are important clinical factors related to the prediction of patients with AUD.

**Conclusion:**

Our proposed FS method could improve the classification performance significantly. It could identify clinical factors related to prediction of AUD from EHRs, thereby effectively helping clinical staff to identify and treat AUD patients and improving medical knowledge of the AUD condition. Moreover, the diversity of features among female and male patients as well as gender disparity were investigated using FS methods and ML techniques.

## Introduction

The term "Alcohol Use Disorder" (AUD) is used to define an uncontrollable level of alcohol consumption. It is correlated with numerous diseases like liver cirrhosis, chronic pancreatitis, upper gastrointestinal cancer, cardiomyopathy, polyneuropathy and dementia, and has a large presence within western countries, especially in Europe [1]. Mid-life AUD mortality rates have increased over the last decades [2]. In Denmark, five percent of all confirmed deaths may be traced to alcohol consumption, which is a typical scenario in many other Western countries as well [3, 4]. Many patients who suffer from AUD will never undergo specialist treatment for their addiction [5]. Machine learning (ML) analysis of electronic health records (EHR) may be used to predict AUD in patients and to help solve this issue. Such an analysis will provide medical staff with observations, allow them to understand the desires of patients and encourage them to discuss how procedures should best be arranged with the particular patient.

One of the most common research areas in the field of clinical data mining is prediction of clinical conditions. It focuses on the role of utilizing ML techniques to classify patients' historical EHRs into one or more predefined clinical groups [6]. It is also a task to identify the target values for a new observation based on a training dataset comprising past observations of known target values. For example, automatically classifying the condition of patients as “AUD-Positive” or “AUD-Negative” (as target values) based on their historical clinical records using ML techniques. However, one of the main problems of developing such a predictive model while dealing with EHR which is the large amount of information that hospitals collect from their patients and the specialized variant of natural language in which it is expressed. EHRs contain a vast amount of information, which increases the number of irrelevant and redundant features. This means that the computational cost of building a classifier using ML algorithms will be substantial.

Feature selection (FS) is one of the steps in the methodology of building a predictive model using ML techniques. In general, FS is a step that removes irrelevant and redundant information to reduce dimensionality. In the FS approach, a small subset of features is selected by minimizing feature redundancy and maximizing feature relevance to the target values. In comparison to feature extration methods that original features are transformed in order to create a feature space of lower dimension than the original feature space [7], in FS, features are selected without transformation from the original feature space and can be used in clinical risk factor discovery. Transformed features have no physical meaning that can be used for deep analysis of the features and clinical factors. Thus, besides the general advantages of FS in reducing problem dimensionality and thereby improving the accuracy of classifiers, FS methods have better readability, interpretability, and explainability in a practical application such as identification of risk factors for clinical conditions [8, 9] (i.e., AUD).

Few studies have aimed to develop ML models to identify patients with AUD and very few studies reported the features, which can be used for identification of patients with AUD. In our previous work [10], we mentioned that variables like family history (FH), psychological and genetic factors are the widely used features in this domain. Demographic features including age, sex, family status, education level, income, occupation, etc. are also widely used in the literature [11]. Features like motives for drinking, drinking behaviors, academic performance, personality, recent depression and anxiety symptoms, and negative life events are employed by Zuba, Gilbert [12] and Bi,Sun [13] for prediction of AUD among college students. Moreover, Kinreich, Meyers [14] considered FH and electroencephalogram signals in their studies predicting the risk of AUD.

Historical (retrospective) data usually cover significant periods of time before appearance of the clinical condition. EHRs contain promising information such as prior presenting symptoms, diagnoses, treatments, length of stays in hospital, etc. that allow researchers to pragmatically study clinical conditions and disorders by identification of clinical factors signaling both the actual condition and also early-onset symptoms and signs. However, computational problems such high dimensionality in EHRs, the heterogeneous nature of EHR datasets and high correlation among features challenge the stability of FS methods [15] toward using ML techniques to identify risk factors and predict patients with AUD.

Although all the above-mentioned studies reported that they successfully achieved their goals, to the best of our knowledge no previous studies have tried to identify relevant clinical factors in prediction of patients with AUD based on the patients’ historical EHR. Such a study may help medical staff to have a better understanding of features with high prediction potential and clinical factors that influence the risk of developing AUD. This is the first study to identify clinical factors and important features relevant to prediction of AUD from historical EHRs.

To address the above-mentioned challenges, the study explores the performance of several filter, wrapper, embedded and ensemble FS trained on historical EHRs in an effort to identify clinical factors and important features for the identification of patients with AUD. The proposed methodological framework will firstly allow us to understand which FS method can produce the best feature subset for the identification of patients with AUD by running ML algorithms on EHRs. Secondly, the selected features will empower medical staff to develop a deeper understanding of the most critical manifestations and clinical factors of AUD.

The remainder of this paper is organized as follow: Section two discusses the proposed method including the characteristics of the historical EHR dataset and the preprocessing techniques used to clean the dataset, description of the FS methods and the experimental setup that is used to evaluate the performance and stability of the FS models. Section three presents the experimental results and discussion, Section four discusses the pespectives, Section five present conclusion, and Section six discusses the limitations and future works.

## Methods

**Fig. 1:**
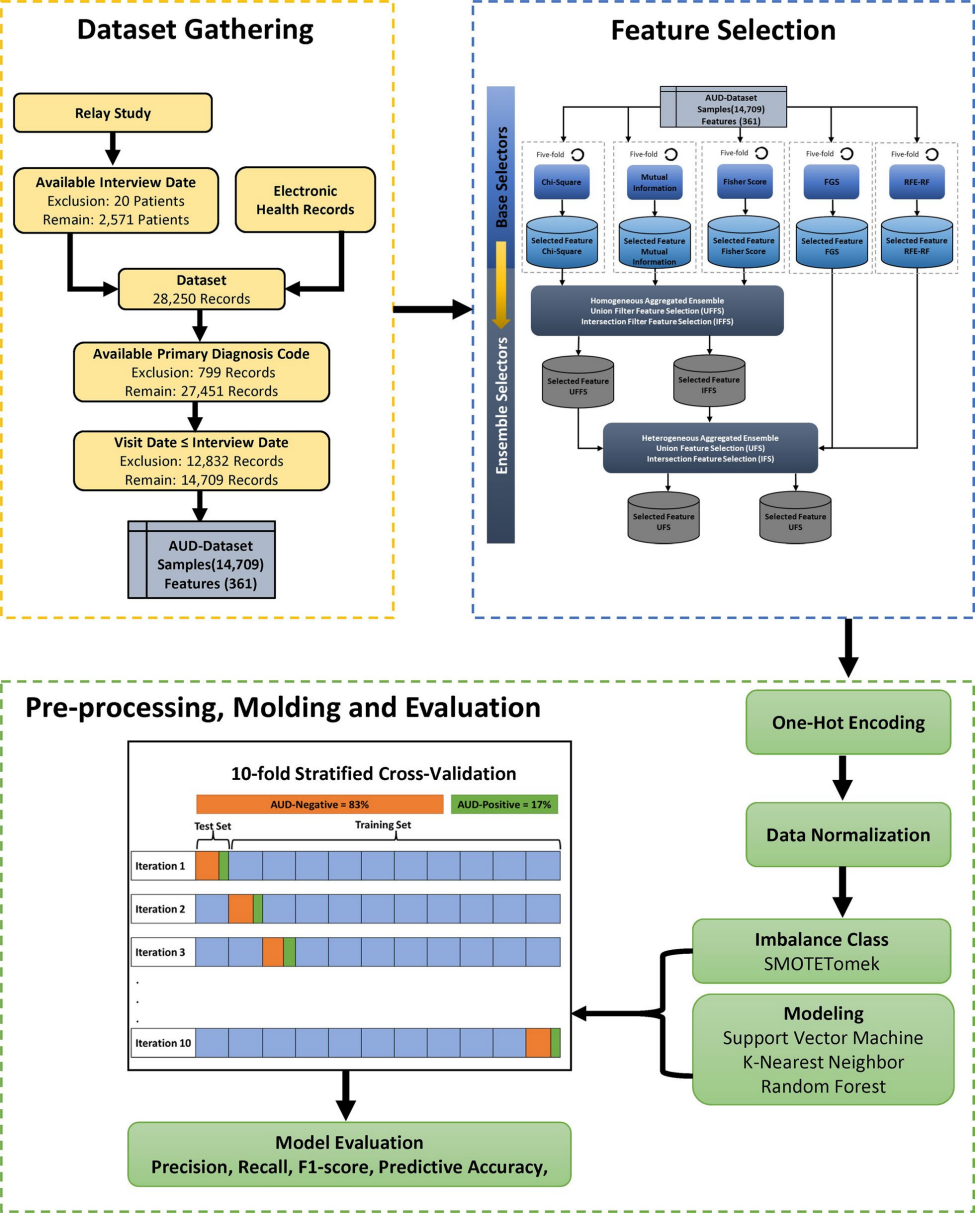
Working diagram of the proposed methods

As it is shown in Fig. 1, the overall methodology proposed for this study encompasses three phases: Data Gathering, Feature Selection, and Pre-processing, Modeling, and Evaluation. In this study, clinical researchers were engaged through all stages of the proposed methodology. For example, besides storing datasets in a secure database, they declared the main idea of labeling patients’ EHRs based on the results of the Relay study. Moreover, medical reasoning about individual clinical factors, specifically the primary diagnosis, was discussed in detail with them over several iterations in the FS phase.

### Data gathering

**Fig. 2:**
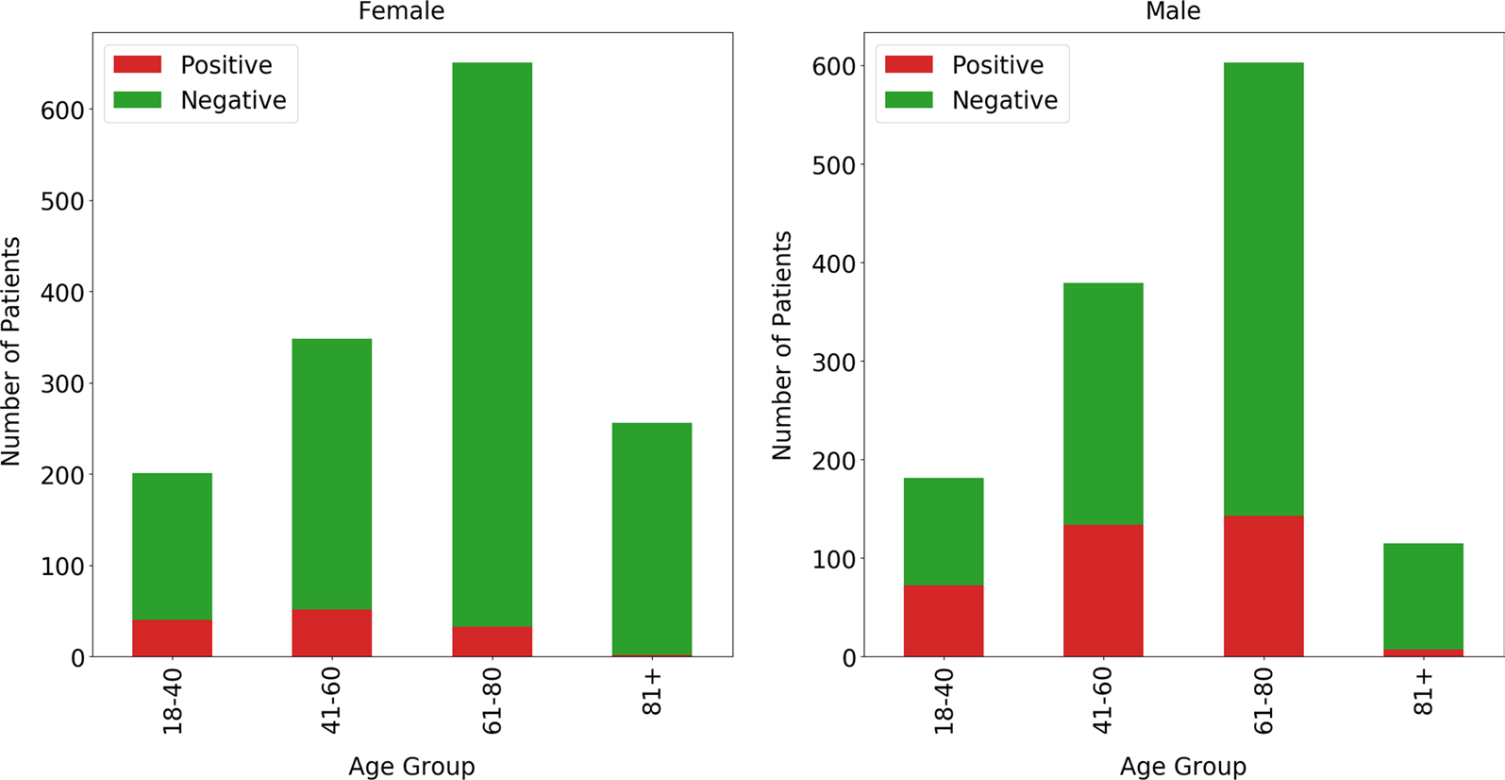
Overview of study population

The study population consists of patients aged 18–101 years hospitalized from January 2012 until June 2016 at Odense University Hospital (OUH) in Denmark for at least 24 h. The data is collected from two sources, the Relay study [16–18] and the EHRs from OUH. The Relay study systematically gathered data from patients admitted to the Gastrointestinal, Neurological, and Orthopedic Departments at OUH over the duration from October 2013 to June 2016. By contributing to a survey based on the Danish edition of the Alcohol Use Disorder Identification Test (AUDIT), patients documented their food, smoking, alcohol, and exercise behaviors. The outcomes of AUDIT vary from 0 to 40; AUDIT results are categorized in AUD positives for those that are above 8 [19]. The findings were 457 AUD-Positive patients and 2114 AUD-Negative patients. In the current study, these groups are used to construct the classification model, as target values of the training dataset. Figure 2 illustrates how our sample data were allocated according to gender, age group and AUD status.

The EHR dataset is the historical medical database of the Relay study participants. This dataset is a collection of reported hospital admissions and referrals to the OUH departments of Gastrointestinal, Neurological and Orthopedic. The dataset includes the patient's personal id number (called CPR number in Denmark), age, gender, length of stay at the hospital, type of admission, interventions, ICD-10 diagnosis codes and health-related conditions from January 2012. This indicates that the data collection consists of the patient's EHR data from 18 months prior to admission to OUH up to their participation in the Relay interview. The CPR number allowed us to link each person's EHR and Relay records. However, for data security reasons and to comply with GDPR, all CPR numbers have been anonymized and stored on a on secure virtual servers operated by Open Patient data Explorative Network^1^ (OPEN) in the Region of Southern Denmark. All clinical records were labeled based on the AUDIT test performance, which is either AUD-Positive (AUDIT score greater than 8) or AUD-Negative (AUDIT score equivalent to or less than 8).

The preparation of the final datasets was preformed based on the inclusion criteria as shown in Fig. 1. This indicates three inclusion criteria including:

1. Patients with available Interview date.
  The Interview date during the Relay Study was the variable which was supposed to be used for identifying the historical records of patients, and it should precede the interview date. Any records having a null value in this variable were dropped from the datasets.
2. Clinical records with a valid ICD-10 code for Primary Diagnosis.
  The Primary Diagnosis is an ICD-10 code that describes the clinical staff’s initial diagnosis of the patient who had been referred to the hospital. This is a key feature that was supposed to be used in this study. Thus, any missing Primary Diagnosis code in the patient’s records also resulted in an elimination of the record.
3. Clinical records preceding the interview time of patients at the Relay Study.

The EHRs contained the clinical records of all patients who attended the Relay Study, dated between January 2012 to July 2016. This, however, does not mean that all the records would indicate the historical EHRs of all the patients. (Imagine a patient attending the Relay study in January 2014). The clinical records indicating the patients' data from January 2014 to July 2016 cannot be considered as historical data, therefore, the interview date stated for the Relay Study, and the Visit Start from the EHRs were considered for the purpose of identifying the historical records of each patient, while the rest of them were eliminated.

Applying the inclusion criteria to the integrated datasets resulted in a selection of 14,709 clinical records of 2571 patients. Among all the patients, 2114 were AUD-Positive while 457 patients were AUD-Positive The final dataset was referred to as the AUD-Dataset in the subsequent descriptions. Therefore, Regarding AUD-Dataset noises, including missed value and weight discrepancies, we agreed to exclude patients that had missing values (e.g., the date of an interview) or noises from the data set. The list, definition and data type of variables in the AUD-Dataset are presented in Table 1.

**Table 1:** Description of variables

Variable	Description	Data type	Feature range
AUD Status	Either positive or negative. They are the target values as well. It is considered as class label	Binary	–
		AUD-Positive = 1	
		AUD-Negative = 0	
Gender	Male or female	Categorical	(*f*_1_, *f*_2_)
Age	Age of patient at time of Relay study	Numerical	(*f*_3_)
Admission Type	Admitted patients or outpatients	Categorical	(*f*_4_, *f*_5_)
Length of Stay	The amount of time the patient spent at the hospital for each visit	Numerical	(*f*_6_)
ED	If the patient visited the emergency department prior to admission	Binary	(*f*_7_, *f*_8_)
ICU	If the patient was transferred to the ICU	Binary	(*f*_9_, *f*_10_)
Action Diagnosis	Reason why patients visited the hospital, scored according to the Danish version of ICD10 codes	Categorical	(*f*_11_, *f*_12_, … *f*_361_)

### Feature selection

In classification, the primary goal of FS is to select a group of strongly discriminant features. This indicates that FS attempts to select features capable of discriminating against samples belonging to various groups and tests the importance of characteristics depending on their potential for distinguishing between different classes. Considering the AUD-Dataset in the form of $N = \left\{ {\left( {f_{i} ,y_{i} } \right)} \right\}$, where *y*_*i*_ is the class label defined by the AUDIT test, $f_{i} \in {\mathbb{R}}^{d}$ is a d-dimensional vector, which consisted by $\left\{ {f_{i1} ,f_{i2} , \ldots ,f_{i361} } \right\}$ feature set. Our goal in this study is to select the most useful feature while *f*_*i*_ is said to be relevant to a class *y*_*i*_*,* if *f*_*i*_ and *y*_*i*_, are highly correlated.

Generally, FS for classification tasks may be divided into filter methods, wrapper methods, and embedded methods [20]. In filter methods, the selected features present general characteristics of the dataset as well as measures (such as, dependency, consistency, distance, information, and correlation) of the features in comparison to the target values, and they are independent of the classifier [21]. The benefit of the independency of the filter methods' FS from the classifier is that the bias of the ML algorithm does not interact with the bias of the FS method [22]. On the other hand, in wrapper methods, the quality of selected features is dependent on the performance of the ML classifier. Wrapper methods utilize a particular ML classifier for searching the subset of features and then evaluate the selected features based on the accuracy of the classifier. This process continues until the best features are selected [23]. This significantly increases the computational cost of the operation for datasets with a high number of features.

In embedded methods, candidate features are first identified using a filter method, and then the best features are selected using a wrapper method depending on the accuracy of the ML model [24]. Therefore, the drawbacks of filter and wrapper methods are addressed by utilizing a hybrid method. Since each method has several advantages and disadvantages, determining which method should be used to select the best features is difficult and dependent upon many factors, such as predictability, interpretability, and computational cost. Therefore, using a combination of filter, wrapper, and embedded methods, "ensemble FS", for selecting features normally produces the most efficient result [25]. The goal of this method is to produce different feature subsets from a training dataset and choose the best features based on the aggregation function.

One of the key difficulties in adapting FS methods to EHRs is the selection of suitable strategies to overcome the high dimensionality problem. The aim is to minimize the number of features in the AUD-dataset, develop an accurate predictive model to identify patients with AUD, and identify clinical factors related to AUD from historical EHRs.

For this purpose, a multilevel FS framework is developed examining the performance of different FS methods in order to achieve the goals. The resulting framework is shown in Fig. 1*(Feature Selection)* as well as Fig. 1 in Appendix I*;* it consists of two operational levels: base selector level and ensemble selector level.

#### Base selector

The first level (base selectors) consists of five individual FS methods that use one of three approaches: filter, wrapper, and embedded. Each of these base FS methods can select features based either on feature ranking or on selecting a subset of relevant features. Since the goal of this study is to identify the clinical factors in AUD as well as feature reduction for prediction of AUD, feature ranking techniques are chosen. These select features from a list of all features, ordered according to their relevance to the event class.

Identifying the impact of each feature on the target values is one of the vital factors. This means that in one of the levels, features must be ranked independently of the feature space. This is the task that can be done by univariate statistical methods in the filter FS layer. However, besides the limitations of filter methods, applying FS methods with similar primary ideas tend to produce similar results. Thus, a wrapper method based on feature subset searching techniques and an embedded method based on pruning techniques are included in our FS framework. This leads us to shaping the second level as homogeneous and heterogeneous aggregated ensemble selector, since in the first level there are same kinds (filters) and different kinds of FS methods.

##### Filter selectors

Filter FS methods usually include two main steps: first, features are ranked or weighted based on some measures evaluated either as univariate or multivariate; and second, the highest-ranked features are chosen as the main features for building the classification model. Features are ranked based on a binary weight in the interval [0, 1] or [− 1, 1], to each feature in which the greatest value is the most relevant feature. The most widely used FS methods in classification tasks [20] are Mutual information (MI) [26], Chi-squared (*chi*^*2*^) [27], and Fisher score (FIS) [28]. These three filter FS methods are selected at this stage because they use different metrics to select features, so the diversity of features will be ensured.

*chi*^*2*^ measures the dependency between a feature *f* and a class *y* and can be contrasted with one degree of freedom to judge extremeness compared to the normal distribution. If the *chi*^*2*^ score is high, it indicates that a feature is likely to be correlated with a class, and therefore the feature is selected for training. The features are ranked according to:

1 $$Chi^{2} = \mathop \sum \limits_{i = 1}^{n} \mathop \sum \limits_{j = i}^{k} \frac{{\left( {A_{ij} - E_{ij} } \right)^{2} }}{{E_{ij} }}$$

where *m* is the number of data records, *k* number of classes, *A*_*ij*_ the number of samples in the *i*th interval, *i*th class (observed frequency), and *E*_*ij*_ is the expected frequency of *A*_*ij*_, and it was calculated as:

2 $$E_{ij} = \frac{{R_{i} . C_{j} }}{N}$$

where *R*_*i*_ is the number of samples in the *i*th interval, *C*_*i*_ the number of samples in the *j*th class, and *N* is the total number of samples.

After calculating the *chi*^*2*^ value of all features, they were sorted in descending order. Considering the degree freedom (DF) $df = \left( {k - 1} \right) \cdot \left( {m - 1} \right)$, the most important features are selected. Since in the AUD-Dataset most of the features are having two intervals, (*k* − 1) was used as the DF for the *chi*^*2*^, which is equal to 1 in this study. Moreover, the significant level for the *chi*^*2*^ is set to be 0.05, which if the value is higher than this, then the feature is significant, and it is selected.

*FIS* is a univariate ranking method that chooses features that give identical values to samples of the same class and diverse values to samples of different classes. With this intuition, the score for the *i*th feature *f*_*i*_ is calculated by the FIS as:

3 $$F_{i} = \frac{{\mathop \sum \nolimits_{k = 1}^{K} n_{j} \left( {\mu_{ij} - \mu_{i} } \right)^{2} }}{{\mathop \sum \nolimits_{k = 1}^{K} n_{j} \rho_{ij}^{2} }}$$

where *μ*_*ij*_ is the mean and *ρ*_*ij*_ is the variant of the *i*th feature in the *j*th class, *n*_*j*_ is the number of records in the *j*th class, and *μ*_*i*_ is the mean of the *i*th feature. We selected the top ranked features by computing a score for each feature independently and then selecting the top ranked features with the highest scores. Although this strategy fails to choose features with relatively low individual scores, it may select features with high scores when they are merged together as a whole. Moreover, since by this approach, we evaluated features individually, FIS could not handle redundancy among features [29].

*Mutual Information (MI)* measures the dependency between variables, i.e., the amount of information one feature can give about another feature [26]. This approach is information theory based method [30] which is essential from the point of view of FS as it offers a mechanism for quantifying the importance of a feature subset with respect to the class. In MI, a feature is relevant if it has a high MI gain index. It is used to measure the dependence between features and class labels. It calculates the information gain (IG) between the *i*th feature *f*_*i*_ and the output vector representing the class label *Y*, as:

4 $$Information\, Gain \left( {f_{i} , Y} \right) = E\left( {f_{i} } \right) - E\left( {f_{i} |Y} \right)$$

where *E*(*f*_*i*_) is the entropy of *f*_*i*_ and *E*(*f*_*i*_|*Y*) is the join entropy of *f*_*i*_ after observing *Y*. Entropy (E) is a measure of a random variable's uncertainty. The chance of an event occurring is linked to the level of uncertainty. High entropy implies that each event has roughly the same chance of occurring, while low entropy implies that each event has a distinct chance of occurring. The E is formally calculated as:

5 $$E\left( { f} \right) = - \mathop \sum \limits_{i = 1}^{n} p\left( {f_{i} } \right)\log_{2} p\left( {f_{i} } \right)$$

Entropy is taken as the probable value of the negative of the logarithm of mass likelihood. Let *f* and *Y* be two random discrete features, as in our case in the AUD-Dataset. The joint entropy of *f* and *Y*, with joint mass probability *P*(*f*_*i*_), *y*_*j*_, is the sum of the uncertainty contained by the two features. Formally, joint entropy is calculated as:

6 $$E\left( {f_{i} |Y} \right) = - \mathop \sum \limits_{i = 1}^{n} \mathop \sum \limits_{j = 1}^{n} p(f_{i} , y_{j} ) . \log_{2} \left( {p\left( {f_{i} ,y_{j} } \right)} \right)$$

Therefore, the best features in the AUD-Dataset are selected based on Eq. 4, which can share the amount of information shared by *f* and *y*.

Considering the main goal of this study, which is to identify the clinical factors in AUD as well as feature reduction for prediction of AUD, and having in mind that the size of the AUD-Dataset, which is not a big dataset, splitting it into a training, test, and validation set would lead us to lose some of the most important and hidden features and clinical factors related to AUD. Therefore, we considered the folding technique in addition to union function to calculate the most important features and clinical factors related to AUD. In each fold of this techniques, one-fifth of the AUD-Dataset was held up, and the filter methods were applied to the remaining folds. Then, collect features from every step stored in a data set and union subsets over all features selected on each step of the folding process selected as the features. This process is applied to each individual filter FS method separately. This technique allowed us to avoid losing hidden features that may not have a high rank but are important while also lowering the risk of bias when compared to applying the methods to only a small portion of the AUD-Dataset (using train, test, and validation split techniques) or applying the methods to the entire AUD-Dataset in a single round.

##### Wrapper selector

Wrapper FS methods are usually based on a search engine for searching a subset of relevant features, a classifier to estimate the performance of the selected subset of features, and finally, a return to the search engine component for the next iteration of the relevant subset of features. This process will result in a set of features with the highest estimated value for training a classifier, and it is called “forward stepwise selection” [31].

**Fig. 3:**
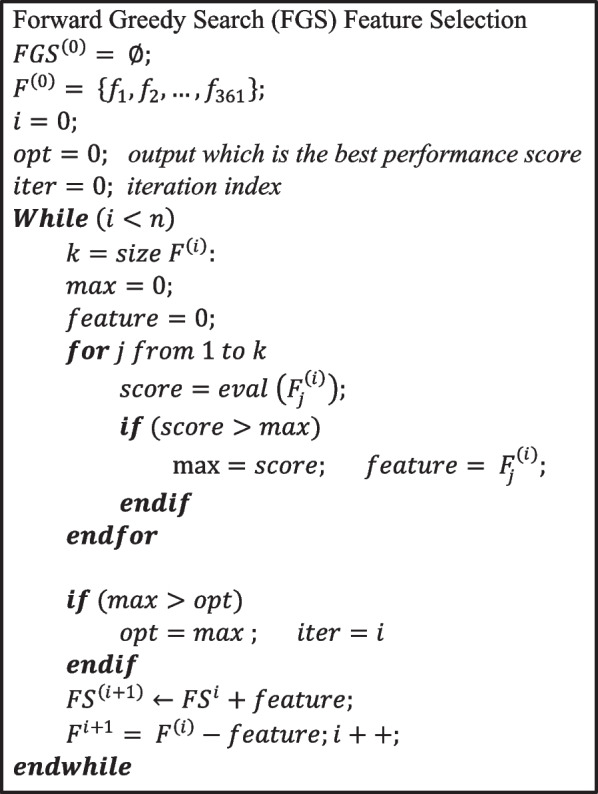
Forward greedy search (FGS) feature selection algorithm

The search in wrapper methods is a crucial step and requires a state space, an initial state, a termination condition, and a search engine [23]. The size of the search space depends on the number of features. In this study, the operation size of the search for 361 features is O(2^361^), which makes it impractical to search the whole space exhaustively. Therefore, we used a forward greedy search (FGS) [32] strategy, which in principle starts with an empty set of features and then at each iteration a new feature which is not included in the current iteration is added independently. At each iteration, the performance metric is stored, and the process is not stopped even if the insertion of the new feature leads to a worse evaluation score. Thus, the overall number of steps is equal to the number of features. The FGS FS, as shown in Fig. 3, begins with an empty feature set and adds one feature that achieves the greatest performance at each iteration.

Since the main task of this process must be done by the search engine, the classifier acts as a black box. In this study, the RF classifier is used as an ensemble classifier to estimate the performance of the feature subsets. Moreover, since the main goal of the search is to find the set with the highest evaluation result, a five-fold cross-validation function is used to evaluate the performance of the selected features. Finally, the evaluation scores through the different iterations are considered, and the overall best result gives the subset of features to be chosen. Nonetheless, the primary problem of sequential forward selection is that it is incapable of removing features that become irrelevant as additional features are added since a score is not defined for each individual feature.

##### Embedded selector

Since wrapper methods utilize a classifier to evaluate the quality of a selected subset of features and since the classifier must be run several times to perform this task, it is a computationally expensive method. To overcome this problem, the search for an optimal subset of features should be programmed into the classifier to combine the subset of features with the classifier learning. This is done by a pruning embedded method, which first utilizes all features to train a predictive ML model, and then tries to eliminate features below the coefficient’s threshold.

In this study, Recursive Feature Elimination [33] using an RF classifier (RFE-RF) is adopted from Chen, Meng [34] study to select the best subset of features. RFE-RF is an embedded FS method based on feature ranking and selection of candidate subsets of features. It recursively builds models by eliminating the features exhibiting dependency and collinearity and builds models with the remaining features until all the features in the AUD-Dataset are used.

**Fig. 4:**
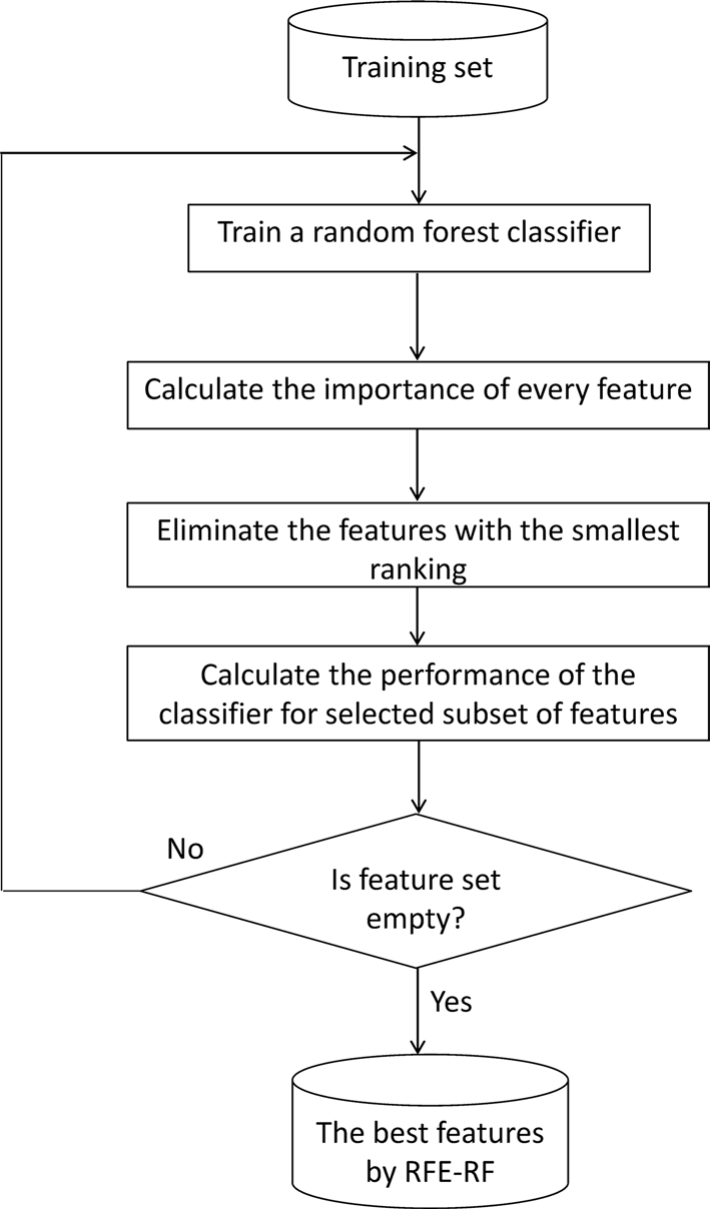
The procedure of the recursive feature elimination (RFE) using random forest (RF) classifier (RFE-RF)

As it is shown in Fig. 4, first the RF classifier is trained on the training set and the importance of each feature is calculated according to its contribution to the classification performance. After that, features are ranked and stored in descending order according to their importance, and the least important feature is eliminated from the list. Then, the remaining features are used to train a new classifier, and the performance of the feature subset for the newly built classifier is calculated. This iterative procedure is continued until the feature set is empty. In the end, there will be a list of classification performances corresponding to each subset of features. A five-fold cross-validation technique is used to estimate the performance of each trained RF classifier, and a list is generated to store the validation score of each candidate feature subset. Finally, the feature subset with the highest accuracy is selected as the optimal feature subset.

#### Ensemble selector

The second level consists of four ensemble FS, two homogeneous, which are the results of the aggregated features according to the same kind of selectors, and two heterogeneous, which are the results of the aggregated features according to different kinds of selectors.

##### Homogeneous selector

There are a variety of approaches to designing an ensemble FS [35]; we focused on the aggregation functions, including union and intersection. In a homogeneous ensemble, the same kinds of FS methods are aggregated based on union and intersection. This means that two different strategies, including union and intersection are considered for aggregation of selected features from MI, *chi*^*2*^, and FIS, as shown in Fig. 1*(Feature Selection)* as well as Fig. 1 in Appendix I. In the first homogeneous aggregated approach which is the Union Filter FS (UFFS) method, all the selected features in Mutual information, *chi*^*2*^, and FIS are used in the union of selected feature sets by them. More formally, the UFFS selects features by:

7 $$UFFS = F_{{\left( {MI} \right) }} \cup F_{{\left( {chi^{2} } \right)}} \cup F_{{\left( {FIS} \right)}}$$

where $F_{{\left( {Mutua\,information} \right) }} ,F_{{\left( {fisher\,score} \right)}}$, and *F*_(*Chi-square*)_ are the set of features which selected by MI, FIS, and *chi*^*2*^, respectively.

The second homogeneous aggregated approach is the Intersection Filter FS (IFFS), which will produce feature sets based on overlapping features that appear in all selected feature sets from filter FS methods. It is formally defined as:

8 $$IFFS = F_{{\left( {MI} \right) }} \cap F_{{\left( {chi^{2} } \right)}} \cap F_{{\left( {FIS} \right)}}$$

##### Heterogeneous selector

By contrast, the heterogeneous ensemble is generated based on aggregation through different kinds of FS methods. Two-stage aggregation approaches, including union and intersection, are employed for heterogeneous ensemble FS, which produces the final subset of features, as shown in Fig. 1*(Feature Selection)* as well as Fig. 1 in Appendix I. The first heterogeneous aggregated approach is the Union method (UFS), which is used to reduce the selected features via RFE-RF, FGS, and UFFS, which is defined as:

9 $$UFS = UFFS \cup IFFS \cup REE - RF \cup FGS$$

The second heterogeneous aggregated approach is the intersection method (IFS), which is used to reduce the number of features based on the features that appear in the overlap between RFE-RF, FGS, UFFS, and IFFS. Due to the disadvantage of FGS which the ranking of features is not specified, we were not able to rank the selected features by heterogenous selectors. The IFS is formally defined as:

10 $$IFS = UFFS \cap IFFS \cap REE - RF \cap FGS$$

As a result, there are four ensemble selectors for performance comparison as well as five single selector methods. There are three subsets based on filter FS (*chi*^*2*^, MI, FIS), one wrapper FS (FGS), one embedded FS (RFE-RF), two ensembles based on filter FS (UFFS and IFFS), and two ensembles based on single FS (UFS and IFS), and the baseline feature subset without FS. Finally, the selected features from the best FS methods are ranked based on the overall mean of their importance and will be present amongst the top clinical factors related to the prediction of AUD using EHRs.

### Data pre-processing modeling and evaluation

#### Data pre-processing

In the AUD-Dataset, the categorical data (see Table 1) was encoded into numerical values, as ML algorithms only recognize number values as variables of input [36]. One of the solutions commonly used to solve this issue is to transform a categorical variable into a series of binary features using a one-hot encoding technique. This method transforms a single variable with n observations and d distinct values into *d* binary features with *n* observations each, where each observation indicates the presence (1) or absence (0) of the dichotomous binary variable. Also, to obtain a common scale for all variables, the traditional normalization technique was used.

As shown in Fig. 2, the AUD-Dataset, consisting of 2114 patients in the majority class and 457 in the minority class, is unbalanced, as shown in Fig. 2. Based on the definition of an imbalanced class distribution proposed by Zhu, Guo [37], the AUD-Dataset was imbalanced in classes where the imbalanced class ratio was 4.9 and the majority rate was 83%. Synthetic Minority Oversampling (SMOTE) [38] is a common sampling approach to deal with an imbalanced class problem. This is a technique in which oversampling of the minority class is accomplished by producing synthetic samples. However, because some majority class samples might be invading minority class space, class clusters in this technique might not be well defined. To overcome this limitation, in this study, we employed SMOTETomek [39], which is a hybrid sampling approach. This approach is a combination of the SMOTE and the Tomek Link method in the form of a pipeline. In this approach, first Tomek Link does the under-sampling of the majority class by eliminating the noises, and then SMOTE does the over-sampling of the minority class to arbitrarily create records and expand minority class records.

#### Data modeling

Using supervised ML (SML) algorithms, the selected features are used to build the AUD classification models. In this study, the SML algorithms learn the classification rules from the selected features. The most commonly employed discriminatory SML algorithms in clinical decision support, i.e., support vector machine (SVM), k-nearest neighbors (KNN), and RF [40, 41], were equipped to analyze all selected features and classify them into two classes, AUD-Positive and AUD-Negative. Since one of the aims of this study is to select features for prediction tasks, it is necessary not to focus on a linear classifier but to attempt to map the data to a higher dimensional space where the classification is more accurate. The performance of SVM differs according to kernel selection, soft margins, and selection parameters. Three parameters were examined: kernel types, the C value, and γ, via an RBF kernel with γ ∈ [0.001, 0.01, 0.1, 1, 10] and C ∈ [0.1, 1, 10]. Moreover, the effectiveness of the KNN relies on the value of *K,* which in this study lies in the range of 1 to 10. RF is an ensemble learning method that classifies a new instance by combining a variety of previously constructed decision trees. In this study, the number of trees is set to be 50 for all RF classifiers. The AUD-Dataset is used to construct the predictive models, using a fivefold cross-validation process to set the hyperparameters of the SVM and KNN classifiers. The best hyperparameter values for each classifier are identified from a set of values. Table 2 shows the final parameters that are used in development of predictive models.

**Table 2:** Hyperparameters of the models

Model	Hyperparameters
Support vector machine	kernel = RBF, *C* = 10, gamma = 0.001
Random forest	Number of DT = 50, max depth = 30
K-nearest neighbor	Number of *k* = 7

#### Model evaluation

Using the receiver operating characteristics curve (ROC) and the area under the ROC curve (AUROC), the area under the precision-recall curve (AUPRC), Precision, Recall, F1-Score, and overall predictive accuracy (ACC), the predictive performances of the constructed classifiers were evaluated. Through seeking values for true positive (TP), false positive (FP), false negative (FN), and true negative (TN), the values of these performance metrics can be determined.

- Predictive Accuracy (ACC)
  The most popular measure of the classifier’s performance is ACC, which evaluates the algorithm's overall effectiveness by calculating the likelihood of the class label's actual value [42]. Measuring the predictive accuracy is the fastest way to understand whether the predictive model has been trained correctly and what the overall performance is. However, it is not the best option to be considered since it cannot give detailed information about the performance of the classifier. The ACC ratio is defined as:
  11 $$Accuracy = \frac{TP + TN}{{TP + TN + FP + FN}}$$
- Precision
  Precision is a performance metric that determines how many of the records that were expected to be positive were truly positive. The main aim of looking at this number is to decrease the number of false positives. Precision can be defined as follows:
  12 $$Precision = \frac{TP}{{TP + FP}}$$
- Recall (True Positive Rate)
  Recall or True Positive Rate (TPR) describes the sensitivity of the classifier. The number of positive samples captured by accurate forecasts is measured by Recall. When all positive samples must be identified, and all false negatives must be avoided, Recall is considered as a performance metric. It is defined as follows:
  13 $$Recall \left( {True\,Positive\,Rate} \right) = \frac{TP}{{TP + FN}}$$
- F1-Score
  The F1-Score is calculated by averaging Precision and Recall. As a result, it shows the performance of the classifier in detecting positive records. This means that the classifier performs best in the positive class if the F1-Score is high. For binary classifications based on imbalanced datasets, F1-Score can be a more appropriate metric to be considered than accuracy.
  14 $$F_{1} \,Score = 2 \times \frac{Precision \times Recall}{{Precision + Recall}}$$
  The AUROC is a single number that measures the total area underneath the ROC curve and thereby summarizes the performance of the classifiers, as long as we assume that FP and FN are equal mistakes. In most medical situations, FN is considered more serious as these people are not identified by the test. Individuals given an FP classification will be tested further, which provides the opportunity to change the classification. ROC curve visualizes the trade-off between TPR and False Positive Rate (FPR) by displaying them for various threshold settings (cutoff points). In particular, the ROC curve attempts to map the cumulative distribution function of a defined probability distribution in the y-axis against the x-axis, for both true and false identified events. In this curve, the y-axis is the TPR, which is defined in Eq. 13, and the x-axis is the FP rate which is calculated as:
  15 $$False\,Positive\,Rate = \frac{FP}{{TN + FP}}$$
  The AUPRC is another widely used performance metric in binary classification problem. It is a threshold-independent measure that estimates the area under a curve formed by a trade-off between several characteristics of performance as the model's prediction threshold changes. In the AUPRC curve, Recall is on the x-axis and Precision is on the y-axis. In imbalanced datasets, such as AUD-Dataset, the AUPRC is more informative than AUROC [43]. The AUPRC is also called the average positive predictive value or the average precision [44].

The performances of all classifiers are evaluated using tenfold cross-validation. The k-fold cross-validation procedure is a frequently used type of evaluation setup that is intended to minimize overfitting in a predictive model, especially in our cases where the quantity of data available is restricted. This approach reduces the level of prediction error deviation; maximizes the usage of data for both train and test without generating overfitting or overlapping testing and validation set; and protects against experimental theory provided by arbitrarily splitting data [45, 46].

In the proposed setting for this study, the cleaned AUD-Dataset is divided into k-equal sizes, and k − 1 AUD-Dataset is coupled for the training of the classifiers, while the kth fold is reserved for testing the classifiers. The classifier validation procedure is done 10 times. This indicates that 90% of the AUD-Dataset is utilized to train the proposed model, while only 10% of the AUD-Dataset is used to test the model. This procedure is repeated k = 10 times and an average result is obtained at the completion. Stratified sampling is utilized during cross-validation to ensure uniform and consistent folds. It should be noted that the SMOTETomek and each ML algorithm are used internally to a stratified cross-validation [47] through a pipeline provided by [48].

## Results and discussion

The research population consisted of 2571 patients aged 18 to 101 years who, between January 2012 and June 2016, had visited OUH at least once. Among all, 457 individuals are categorized as AUD-Positive, and 2114 are categorized as AUD-Negative, and about 46% of patients are male. Throughout the entire data collection span, 14,079 health records with at least one record per patient were recorded. For an AUD-Negative patient, the longest hospital stay was 5574 min (a 63-year-old male). The baseline characteristics of patients and the AUD-Dataset are presented in Table 3. As it is mentioned in the previous chapter and presented in Table 1, there are 361 features, including 530 different ICD-10 codes, given as the action diagnosis (AD) amongst all records. The most commonly used ADs were DM16 (Coxarthrosis (arthrosis of hip), 902 occurrences), DM17 (Gonarthrosis (arthrosis of knee), 842 occurrences), DT84 (Complications of internal orthopaedic prosthetic devices, implants and grafts, 597 occurrences), DI63 (Cerebral infarction, 496 occurrences) and DM19 (Other arthrosis, 422 occurrences) in the AUD-Dataset. The top 20 AD in the AUD-dataset can be seen in Table 4. Using a one-hot-encoding technique to convert all categorical variables to binary variables generated 361 features. Variables such as gender and main hospital departments were also converted to binary numbers. Considering all the variables described in Table 1, 361 features from the pre-processed AUD-Dataset are used in selecting the best subset of features for prediction of AUD. The two groups of FS methods, the number of features for each FS method, and the accuracy of the classification models are presented in Table 5.

**Table 3:** Characteristics of study population

Variable	AUD positive		AUD negative	
	n	%	n	%
Patients	457	17.7	2114	82.3
Gender				
Female	122	4.7	1256	48.8
Male	335	13.2	858	33.3
Age				
18–40	109	4.2	260	10
41–60	174	6.7	507	19.7
61–80	165	6.4	1007	39.1
81 +	9	0.3	340	13.2
Clinical records	2366	16.8	11,713	83.2
ED	214	1.51	744	5.2
ICU	26	0.2	15	0.1

**Table 4:** Frequency of top 20 action diagnosis among all patients

No	Action diagnosis	Description	Freq
1	DM16	Coxarthrosis	902
2	DM17	Gonarthrosis	842
3	DT84	Complications of internal orthopaedic prosthetic devices, implants and grafts	597
4	DI63	Cerebral infarction	496
5	DM19	Other arthrosis	422
6	DS72	Fracture of femur	406
7	DK50	Crohn disease	390
8	DZ03	Medical observation and evaluation for suspected diseases and conditions	348
9	DR29	Other symptoms and signs involving the nervous and musculoskeletal systems	347
10	DK51	Ulcerative colitis	337
11	DS82	Fracture of lower leg, including ankle	330
12	DQ65	Congenital deformities of hip	297
13	DI69	Sequelae of cerebrovascular disease	275
14	DS42	Fracture of shoulder and upper arm	275
15	DK70	Alcoholic liver disease	269
16	DK91	Postprocedural disorders of digestive system, not elsewhere classified	247
17	DS52	Fracture of forearm	240
18	DT93	Sequelae of injuries of lower limb	240
19	DK86	Other diseases of pancreas	238
20	DT92	Sequelae of injuries of upper limb	223

**Table 5:** Number of selected features and influence of each feature selection method on evaluation metrics

FS method	Number of selected features	Machine learning method																	
		Support vector machine-RBF						K-nearest neighbor						Random forest					
		P	R	F1	Acc	AUROC	AUPRC	P	R	F1	Acc	AUROC	AUPRC	P	R	F1	Acc	AUROC	AUPRC
*Base selectors*																			
Baseline	361	0.62	0.30	0.41	0.80	0.69	0.40	0.69	0.49	0.57	0.86	0.86	0.54	0.77	0.65	0.71	0.90	0.93	0.96
Chi^2^	106	0.83	0.41	0.55	0.88	0.81	0.55	0.72	0.59	0.65	0.89	0.89	0.65	0.87	0.70	0.77	0.93	0.95	0.77
MI	172	0.75	0.43	0.54	0.87	0.76	0.54	0.67	0.50	0.57	0.87	0.88	0.57	0.77	0.64	0.70	0.90	0.94	0.70
FIS	107	0.78	0.44	0.56	0.88	0.81	0.56	0.69	0.71	0.76	0.88	0.89	0.65	0.82	0.71	0.76	0.92	0.95	0.76
FGS	66	0.74	0.36	0.48	0.87	0.79	0.48	0.68	0.52	0.59	0.87	0.89	0.59	0.79	0.71	0.75	0.92	0.95	0.48
RFE-RF	131	0.79	0.64	0.71	0.91	0.90	0.66	0.79	0.74	0.76	0.92	0.95	0.74	**0.83**	**0.85**	**0.84**	**0.94**	**0.97**	**0.83**
*Ensemble selectors*																			
IFFS	58	0.73	0.33	0.45	0.86	0.74	0.45	0.64	0.53	0.58	0.87	0.82	0.57	0.74	0.61	0.67	0.88	0.91	0.66
UFFS	**223**	0.81	0.56	0.66	0.87	0.90	0.66	0.75	0.73	0.74	0.94	0.91	0.74	**0.87**	**0.81**	**0.84**	**0.94**	**0.98**	**0.83**
IFS	19	0.82	0.17	0.27	0.85	0.62	0.27	0.49	0.47	0.48	0.82	0.79	0.48	0.75	0.36	0.49	0.87	0.89	0.48
UFS	**272**	0.81	0.65	0.72	0.91	0.91	0.72	0.80	0.75	0.77	0.92	0.95	0.77	**0.89**	**0.91**	**0.90**	**0.96**	**0.98**	**0.89**

Bold values indicate better predictive performances as well as a better subset of features

*Precision (P), Recall (R), F1-Score (F1), Accuracy (ACC), Area Under the Receiver Operating Characteristic (AUROC), Area Under the Precision-Recall Curve (AUPRC), Mutual information (MI), Chi-squared (chi2), Fisher score (FIS), Forward Greedy search (FGS), Recursive Feature Elimination using a Random Forest (RFE-RF),Union Filter FS (UFFS), Intersection Filter FS (IFFS), Union FS (UFS), Intersection FS (IFS)*

### Base feature selection

The first part of Table 5 shows the effect of the chosen feature selection methods on the classification performance of SVM, KNN, and RF. As seen, the baseline, which contains all 361 features, has the worst prediction accuracy-only 80% using the SVM classifier. In contrast, the RFE-RF embedded method with its 131 features could achieve 89% accuracy with SVM and 93% using RF. Among all filter FS methods, only MI did not reduce the features as much as the other base FS methods. MI reduced the number of features from 361 to 172, which resulted in a slight improvement in classification performance. The *chi*^*2*^ and FIS reduced the number of features from 361 to 106 and 107 respectively., however, none of them could improve the classification performance. In terms of the number of selected features, FGS as the wrapper method reached a maximum accuracy of 88% in the 66^th^ iteration using the RF classifier. This means that the best accuracy was recorded when 66 features were selected using this method. This amount of elimination by FGS resulted in the worst classification performance among base selectors, lower than baseline using the RF classifier. This is most likely due to ‘over selection’ in which too many informative features are eliminated. Overall, RFE achieved the best results among all base FS methods, both in terms of the low number of selected features as well as the accuracy. As can be seen in Table 5, RFE reduced the number of features to 131 and still achieved better accuracy than the other classifiers.

### Ensemble feature selection

The second part of Table 5 shows the influence of each ensemble FS method on the classification performance of SVM, KNN and RF. As can be seen, the performance of the ensemble FSs based on the union of feature sets perform better than those based on intersection. In particular, the best classification model is the result of UFFS method, which achieved a classification accuracy of 93% based on the RF classifier. This method is made by union of the filter FS methods (MI ∪ *chi*^*2*^ ∪ FIS), which reduced the number of features from 361 to 276 features. In terms of feature reduction, IFS reduced the number of features to 27, however, with reduction of too many features it resulted in ‘*over selection*’ and the worst accuracy performance among all models and even below the baseline accuracy of 80% for the SVM classifier. The results indicate that ensemble feature selection through the union aggregation method is unlikely to cause over selection problems, with the least number of features being eliminated leading to a good final classification performance. By contrast, using the intersection combination method eliminates too many features, thereby causing over selection problems, and consequently decreasing the final classification performance.

### Classification model performance

Accuracy is usually considered the most important technique for evaluating ML algorithms. As mentioned above, we used three classifiers to compare the performance of the proposed FS method. As can be seen in Table 5, the accuracy of Baseline models is 80%, 86%, and 90% for SVM-RBF, KNN, and RF classifiers, respectively, with 361 features. Looking at the accuracy of all classifiers based on the nine developed FS methods, the RFE-RF as a based selector, IFFS and UFS as ensemble selectors demonstrated an excellent accuracy of 94%, 94%, and 96% for the RF classifier, respectively. The results of the methods on the KNN classifier also show that the RFE-RF as a based selector, IFFS, and UFS as ensemble selectors achieved the best performance among all other methods.

As we mentioned earlier, Recall or TPR, is an important performance evaluation matrix that describes the sensitivity of the classifier. Recall is important because it shows the AUD-Positive patients are accurately classified. As it is shown in Table 5, the Recall scores for the Baseline classifiers are very poor, with 30%, 49%, and 65% for SVM-RBF, KNN, and RF classifiers, respectively. A very poor Recall score (just over 17%) has been generated and was obtained with the SVM-RBF classifier based on the IFS selector, while the RF achieved the highest recall score (92%) when applied to the UFS 272 features. RF also had Recall score of 85% and 81% based on REF-RF and UFFS respectively. Among the KNN models, the best Recall scores were also achieved based on the features selected by UFS, UFFS, and RFE-RF for scores of 75%, 73%, and 74%, respectively.

**Fig. 5:**
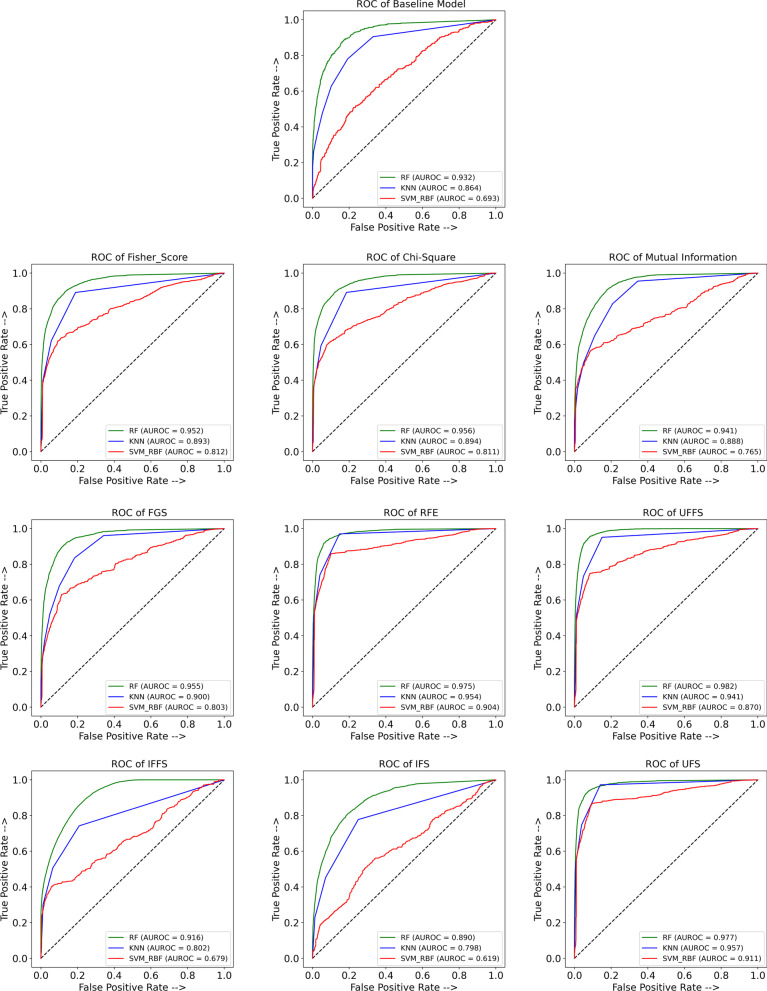
Result ROC curve of random forest (RF), support vector machines (SVM), and k-nearest neighbors (KNN) based on selected features using base feature selection methods including Chi-square, Fisher-score, mutual information, recursive feature elimination (RFE), and forward greedy search (FGS), and based on selected features using ensemble feature selection methods including union filter feature selection (UFFS), intersection filter feature selection (IFFS), union feature selection (UFS), and intersection feature selection (IFS), as well as baseline model

Figure 5 shows the ROC curves from each classifier trained on the result of each FS methods and the full feature set (Baseline) and reports the AUROC in each case. In ML, ROC curves are used to validate the accuracy of predictive models by representing the TP rate versus the FP rate [49]. In dichotomous diagnostic tests (positive and negative tests), ROC plays a vital role by measuring the inherent validity of a test based on TP and FP rate [50]. The AUROC gives an effective measure that represents the area under the ROC curve, and it is a way to summarize the quality of a diagnostic models’ performance. In the literature, an AUROC between 0.9 to 1.0 is regarded as excellent [51]. As can be seen in Fig. 5 (Baseline), the AUROC for SVM-RBF classifier trained by baseline features was 0.679 which is the worst result. The RF classifier used on the RFE-RF output achieved an AUC value of 0.975 (Fig. 5, ROC of RFE), which is the best among all base FS models. On the other hand, the RF classifier for the UFFS method, which trained by the selected features from our proposed ensemble methods, achieved an AUC of 0.982, which is the best performance among all classifiers. In contrast, the SVM-RBF classier which trained by selected features from the IFS model achieved an AUC rate of 0.624 which is the worst performance in comparison to other classifiers, which are trained by features from ensemble FS methods.

**Fig. 6:**
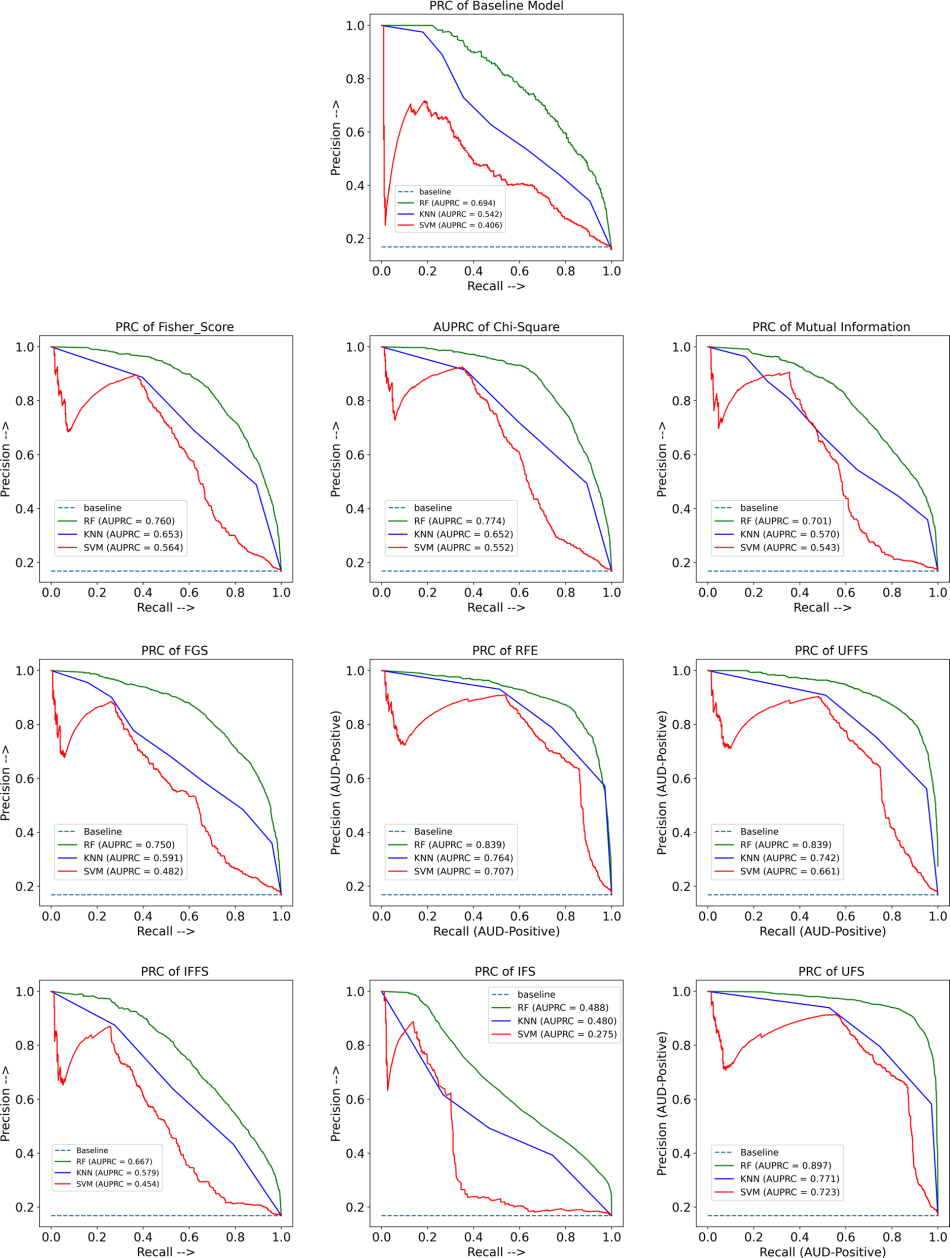
Result precision-recall curve (PRC) of random forest (RF), support vector machines (SVM), and k-nearest neighbors (KNN) based on selected features using base feature selection methods including Chi-square, Fisher-score, mutual information, recursive feature elimination (RFE), and forward greedy search (FGS), and based on selected features using ensemble feature selection methods including union filter feature selection (UFFS), intersection filter feature selection (IFFS), union feature selection (UFS), and intersection feature selection (IFS), as well as baseline model

Figure 6 shows the Precision-Recall curves from each trained classifier trained based on the selected features by the FS methods as well as the full feature set (Baseline Model). It should be noted that the baseline of AUPRC (the light blue dotted line in each curve) is equal to the fraction of AUD-Positives. Since the AUD-Dataset consists of about 17% AUD-Positive and about 83% healthy examples, the baseline AUPRC is 0.17. As it is shown in Fig. 6, the best models were trained based on the selected features through our proposed UFS method. Considering 272 features selected by UFS, a notable result of over 89% was obtained for AUPRC with the RF model, which is the highest among all developed models. When RF was applied to the 131 features of RFE-RF and the 223 features of UFFS, both classifiers achieved an AUPRC of approximately 84%, which is the second-best performance among all. KNN also obtained a good AUPRC score of 77%, 76%, and 74% for UFS, RFE-RF, and UFFS, respectively. Among all the developed models based on SVM-RBF, only RFE-RF and UFS could achieve a reasonable AUPRC score of 70% and 72%, respectively. One of the most widely used and relatively new predictive performance metrics to evaluate the performance of classifiers in the field of medical research is the precision-recall curve, which represents the Recall versus Precision for all possible thresholds. Several studies suggest that the AUPRC is more informative than the ROC curve and AUROC for evaluating the risk model’s prediction performance for an imbalanced class distribution [52], such as in this study where the AUD-Positive rate is low. Since the AUPRC is the area under the curve of the plot of Precision versus Recall across thresholds, and Precision is based on the records that were expected to be AUD-Positive and were truly AUD-Positive, it does not incorporate the number of TN.

In this section, we evaluated the performance of ML algorithms before and after providing a solution to the high dimensionality issue by examining various FS methods. Each FS method, as explained in "Feature Selection" section, focuses on a special metric in order to reduce the number of features and improve the predictive accuracy. Along with AUROC and AUPRC, the performance of models was evaluated based on Precision, Recall, and F1-Score. Our results show that in terms of classification performance, 131 features selected by the RFE-RF among the other base selectors could achieve the best performance when trained with RF. Among the proposed ensemble selectors, the UFS and UFFS methods performed the best when trained with the RF classifier. Overall, the predictive performance of all models, except those based on FGS and IFS, improved in comparison to the Baseline models based on all features. Considering the predictive preferences and disadvantages of RFE and FGS [53], such as computational costlier than filter methods, complexity, greater execution time, and etc., our proposed UFFS method can be considered as the best FS method among all those that were used in this study.

### Clinical risk factors

As can be seen in Table 5, UFFS significantly reduced the number of features from 361 to 233, and it could likewise improve the classification performance. The features selected by UFFS are ranked based on their importance, and the top 20 are presented in Table 6. RF can rank features based on their Gini index (GI) [54] in which higher values indicate more important features. It was found that age, gender, and length of stay at the hospital are the most important factors for the identification of individuals with AUD. In terms of ADs, DK50 (Crohn disease), DK86 (Other diseases of pancreas), DK70 (Alcoholic liver disease), DZ03 (Encounter for medical observation for suspected diseases and conditions ruled out), and DS82 (Fracture of lower leg, including ankle) are the top five clinical factors. DM19 (Another arthrosis), D74 (Hepatic fibrosis), DM16 (Osteoarthritis of hip), DK29 (Gastritis and duodenitis), DT93 (Sequelae of injuries of lower limb), and DI69 (Sequelae of cerebrovascular disease) are ADs with the same GI in the list of important features. Comparing their ranks (Table 6) with their frequency among all patients (Table 4), it can be seen that although DM16 has the highest frequency among all diagnoses, its impact on the final predictive model is not that high. This is due to its frequency among AUD-Positive patients, which is much lower compared to AUD-Negative. Some clinical records with ICD-10 labels, such as DZ03 "Medical observation and evaluation for suspected diseases and conditions" have a high impact on our final predictive model but may have no informative value for medical staff. In the literature, risk factors such as gender and age have been discovered in many studies [14, 55]. However, no studies looked for clinical and risk factors for AUD from EHRs. Our findings show that, based on the AUD-Dataset, diseases in the group of digestive organs, bones, muscles and connective tissue, and nervous systems seem to be highly correlated with the prediction of patients with AUD.

**Table 6:** Top 20 important features extracted by RFE-RF, ranked by GI

No	Feature	Description	Ranking
1	Age	Age at the relay study	0.411
2	LOS	Length of stay at the hospital	0.093
3	Male	Gender	0.037
4	Female	Gender	0.034
5	DK70	Alcoholic liver disease	0023
6	DK50	Crohn's disease	0.013
7	DK86	Other diseases of pancreas	0.012
8	DZ03	Encounter for medical observation for suspected diseases and conditions ruled out	0.01
9	ED	Visitor of Emergensy Department	0.01
10	DS82	Fracture of lower leg, including ankle	0.009
11	Admission	Admited to the hopspital	0.009
12	Outpatient	Ambulatory care	0.009
13	DM19	Another arthrosis	0.008
14	DK74	Hepatic fibrosis	0.008
15	DM16	Osteoarthritis of hip	0.008
16	DK29	Gastritis and duodenitis	0.008
17	DT93	Sequelae of injuries of lower limb	0.008
18	DI69	Sequelae of cerebrovascular disease	0.008
19	DK26	Duodenal ulcer	0.007
20	DG56	Mononeuropathies of upper limb	0.007

### Gender disparity

Historically, alcohol consumption seems to have been a male-dominated behavior, with males drinking more alcohol and inflicting more alcohol-related harm on themselves and others than females [56]. In 2016, a report showed that three million deaths caused by AUD comprised 77% of male [57]. This is because female drinkers consume around one-third of the total quantity of alcohol consumed than male drinkers each year [57]. It was also reported that more female had registered driving under the influence of alcohol in 2017 [56]. Nevertheless, the AUD disparity between males and females is narrowing [56]. The growing prevalence of AUD among females has been a cause for worry in recent years, since females have been exposed to the negative health and behavioral consequences of alcohol consumption sooner and at a lower rate than males [58]. There were various factors that differentiated AUD by gender. Generally, females were smaller than males, with a lower total body water level and a greater total body fat percentage. As a result, alcohol is absorbed more quickly by female’s bodies. Females’ blood alcohol consumption also increases more rapidly and stays higher for a longer period of time than males’ [59]. Furthermore, McCaul, Roach [59] highlighted that gender dissimilarities exist in brain structure, neurochemistry, and function.

Thus, it is necessary to investigate the fact that gender disparity should be taken into account when building predictive models for the identification of patients with AUD. In this regard, we extended our analysis to compare the performance of the predictive models and the diversity of features between female and male patients. First, the AUD-Dataset were divided into two different datasets based on the gender of patients. The other preprocessing steps were inherited from "Data Gathering" section. Our proposed UFFS FS method, which was described in "Homogeneous Selector" section, was then employed to reduce the number of features in the AUD-Dataset. After that, three ML algorithms, including SVM, KNN, and RF, were trained and evaluated based on the methods in "Data Pre-Processing Modeling and Evaluation" section for each group of gender separately.

**Fig. 7:**
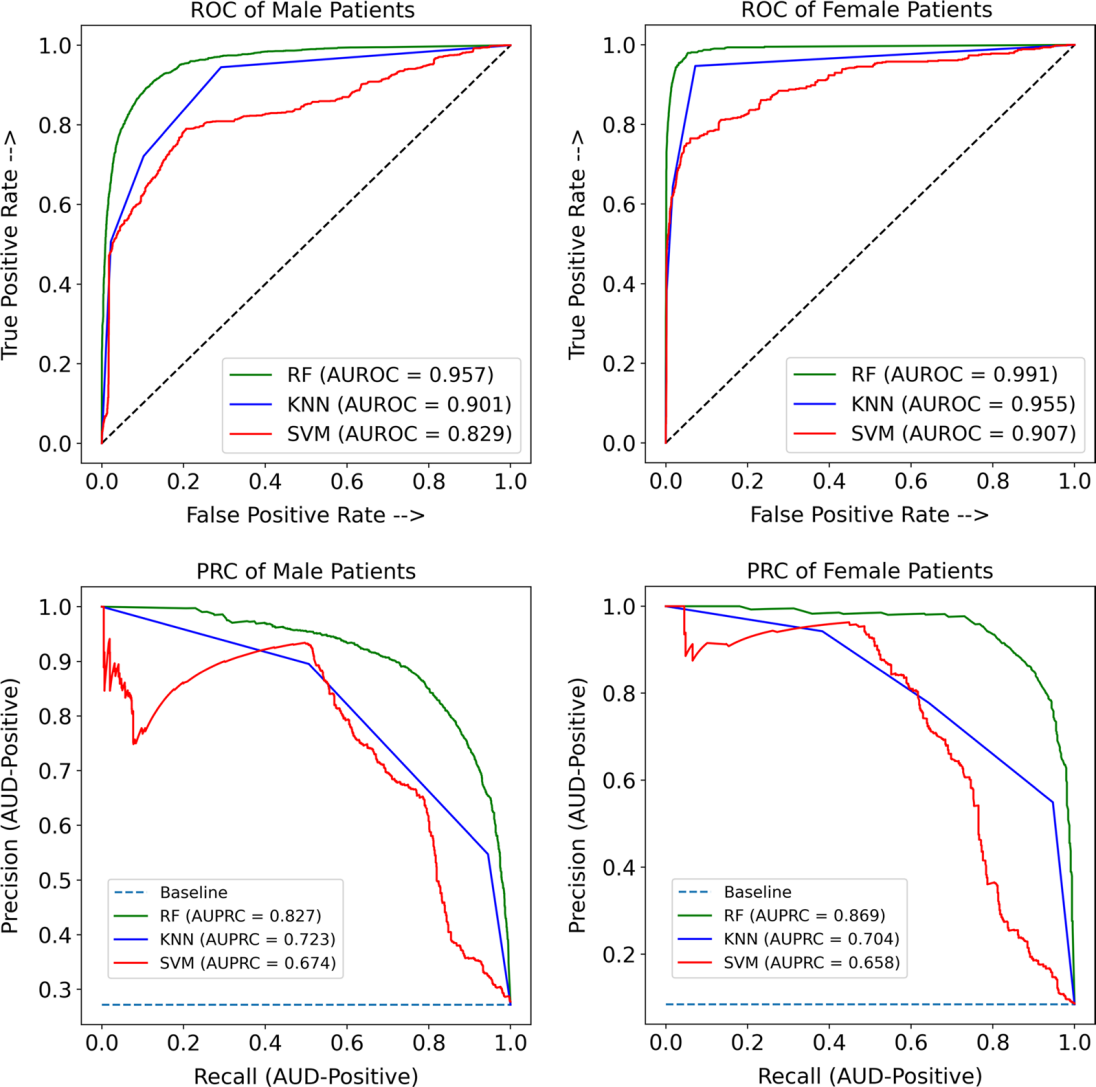
Result ROC and precision-recall curve of random forest (RF), support vector machines (SVM), and k-nearest neighbors (KNN) based on two dataset female patient and male patients

As can be seen in Table 7, our proposed UFFS FS methods could reduce the number of features from 359 to 218 and 233 for Female and Male patients, respectively. In terms of predictive accuracy, RF achieved the best accuracy in both datasets, whereby it achieved 97% and 90%, respectively, for Female and Male patients. Overall, the trained models for Female patients had better accuracy than Male patients. Figure 7 shows the ROC and Precision-Recall curves of the classifiers which were trained for each group of patients based on the selected features. This also indicated that Female patients’ classifiers had achieved better performance than Male patients with AUROC and an AUPRC of 0.99 and 0.88, respectively, for the RF classifier.

**Table 7:** Number of feature and overall accuracy of models

Gender	Number of features	Machine leaning methods											
		Support vector machine				K-nearest neighbor				Random forest			
		P	R	F1	ACC	P	R	F1	ACC	P	R	F1	ACC
Female	From 359 to 218	0.88	0.52	0.66	0.95	0.78	0.64	0.70	0.95	0.91	0.83	0.87	0.97
Male	From 359 to 233	0.81	0.57	0.67	0.84	0.76	0.69	0.71	0.85	0.81	0.82	0.82	0.90

The important features of each group of patients which were selected by the UFFS FS methods were then ranked based on their GI, as noted by the RF classifier. This is presented in Tables 8 and 9 for Male and Female patients, respectively. The results indicated that apart from Age and Length of Stay, the ranks of other features were very different. In terms of AD among the group of Male patients, DK86 (Other diseases of pancreas), DK50 (Crohn's disease), K70 (Alcoholic liver disease), DG56 (Mononeuropathies of upper limb), and DK29 (Gastritis and duodenitis) were identified as the top five clinical factors. In comparison, the top five AD among Female patients were DK70 (Alcoholic liver disease), DK74 (Hepatic fibrosis), DA41 (Other sepsis), DZ03 (Observation for suspected tuberculosis), and DI63 (Cerebral infarction due to thrombosis of precerebral arteries). Among the top 20 important features, four Ads, including DK50 (Crohn's disease), DK70 (Alcoholic liver disease), DM19 (Another arthrosis), and DS82 (Fracture of lower leg, including ankle) were noted among Male and Female patients. Considering Age, Length of Stay, Admission type (Outpatient and Admission), and Emergency Department as other common features among the Female and Male patients, we then deduced that more than 50% of high ranked features were different in the two groups of patients.

**Table 8:** Top 20 important features extracted from male patients

No	Feature	Description	Ranking
1	Age	Age at the Relay study	0.456
2	LOS	Length of stay at the hospital	0.121
3	DK86	Other diseases of pancreas	0.015
4	ED	Visitor of Emergency Department	0.014
5	DK50	Crohn's disease	0.013
6	DK70	Alcoholic liver disease	0.011
7	Outpatient	Admitted to the hospital	0.011
8	Admission	Ambulatory care	0.011
9	DG56	Mononeuropathies of upper limb	0.009
10	DK29	Gastritis and duodenitis	0.008
11	DG40	Epilepsy	0.008
12	DI61	Intracerebral haemorrhage	0.008
13	DG20	Parkinson disease	0.007
14	DK26	Duodenal ulcer	0.007
15	DM19	Another arthrosis	0.007
16	DT93	Sequelae of injuries of lower limb	0.007
17	DT92	Sequelae of injuries of upper limb	0.007
18	DR29	Other symptoms and signs involving the nervous and musculoskeletal systems	0.007
19	DS46	Injury of muscle and tendon at shoulder and upper arm level	0.007
20	DS82	Fracture of lower leg, including ankle	0.007

**Table 9:** Top 20 important features extracted from female patients

No	Feature	Description	Ranking
1	Age	Age at the relay study	0.481
2	LOS	Length of stay at the hospital	0.094
3	DK70	Alcoholic liver disease	0.037
4	DK74	Hepatic fibrosis	0.021
5	DA41	Other sepsis	0.020
6	ED	Visitor of Emergency Department	0.016
7	DZ03	Observation for suspected tuberculosis	0.014
8	Outpatient	Admitted to the hospital	0.014
9	DI63	Cerebral infarction due to thrombosis of precerebral arteries	0.013
10	Admission	Ambulatory care	0.012
11	DS82	Fracture of lower leg, including ankle	0.010
12	DI69	Sequelae of cerebrovascular disease	0.010
13	DK90	Intestinal malabsorption	0.010
14	DS53	Dislocation, sprain and strain of joints and ligaments of elbow	0.008
15	DK76	Other diseases of liver	0.007
16	DM19	Another arthrosis	0.007
17	DM24	Loose body in joint	0.007
18	DK50	Crohn disease [regional enteritis]	0.007
19	DK21	Gastro-oesophageal reflux disease	0.007
20	DS62	Fracture of first metacarpal bone	0.007

Women with AUD-Positive had been given fewer considerations than man, to some extent, because women were less exposed to AUD risk factors [58]. Although the frequency of AUD-Positive was lower in women, as shown in Fig. 2, it has been well proven that women experience more serious biopsychosocial effects of both short-term and excessive heavy alcohol usage than men [58, 60, 61]. Moreover, women progressed faster, from being drinkers to being alcohol dependence [62]. They also faced higher rates of internalizing disorders when compared to men [63]. In their study, Chang [64] noted that there was a lack of an alcohol screening tool with high specificity and sensitivity for pregnant women. There were a lot more evidence which showed the need to have a separate identification test for each group of gender [59]. In this study, we also showed that the clinical risk factors related to prediction of AUD among Female and Male patients were different, based on their contributions to the RF models. Results indicated that the developed models performed with better predictive accuracy for Female patients than Male patients.

## Perspectives

Problematic alcohol use has a high impact on the individuals’ health [65–67] and mortality [3]. The first step in preventing diseases and deaths attributable to alcohol is identifying and acting on the signs. At least in Denmark, identification of patients with alcohol use disorder may be problematic [68]. Hospital admissions form a logical opportunity for acting on signs of alcohol use that directly or indirectly have an impact on the health of the patient. Between 17 and 25% of hospitalized patients drink above the limits recommended by the health authorities or screen positive for problematic use [69, 70], and when problematic alcohol use is not addressed, patients are shown to have high a risk of developing further alcohol-related conditions [71]. It is, however, also well-known that alcohol problems are stigmatized [72] and that healthcare staff are reluctant to ask patients about their alcohol habits [73]. Furthermore, this view of problematic alcohol use may influence the focus and care of this patient group [74]. Reasons for not addressing problematic alcohol use are many and include lack of time and uncertainty about what to look for [75]. Thus, using the presented models as the basis for the development of a decision support system (DSS) for signs of AUD that:

- works without adding time-consuming additional screening procedures to the staff’s workload
- is not influenced by the staff’s subjective attitude
- is grounded in data that is already present in the EHR
- can predict AUD among patients with a high level of Precision could have a huge impact on public health.

A DSS can make it easier for medical staff to identify and act: to talk with the patients already identified to be at risk about what they need to do to recover and stay healthy. Ideally, when a patient attends the emergency department, is admitted to hospital, or is seen in an outpatient consultation, the DSS will alert the staff if the information already stored in the EHR indicates that alcohol may be a complicating factor to the current situation. An alert may be the information needed for the staff in order to address the issue of alcohol where relevant, in particular since the patients themselves are normally positive towards discussing their alcohol use with health care professionals [75]. Such knowledge will enable medical staff to talk to the patient at risk about drinking habits and provide the patients with information on how prognosis can be improved by reducing the intake of alcohol, and referral to relevant treatment if needed. This could be useful not only for hospital staff but also for general practitioners.

In addition to a potential DSS that helps hospital staff to address and intervene in relation to alcohol problems where relevant, our study has further perspectives. Transference of the technology and methods from the present study also have obvious potential in a range of similar conditions where screening of EHR data helps staff to predict clinically-relevant events or complications that may be prevented or mitigated if the staff reacts to early signs. Although this field is relatively new and data on clinical, workload, and efficiency outcomes are sparse, DSSs seem to improve the targeting of health care measures such as preventive services, clinical studies, and therapies [76].

## Conclusion

The large amount of information about patients in EHRs causes dimensionality challenges for ML studies and difficulties in the identification of clinical factors. Clinical factor discovery is important to the study of addictions such as AUD. Dimensionality reduction has been an important and challenging step toward having a viable and accurate ML-based application. FS is the task of removing irrelevant and redundant information from the dataset, and it can be done in many ways. FS for classification handles high dimensionality by selecting the most relevant subset of features to the target value. Besides feature reduction, a powerful FS method can suggest important clinical risk factors related to disorders like AUD. Our study presented a multilevel FS framework which consists of the two operational levels, base and ensemble selectors, which aims to:

- Reduce the high dimensionality in an EHR dataset.
- Develop several predictive models to detect patients with AUD and compare their performance.
- Identify clinical factors related to prediction of AUD from a historical EHR dataset collected from AUD-Positive and AUD-Negative patients of OUH.
- Investigate the diversity of clinical factors among female and male patients and gender disparity based on ML techniques.

The base selector level consists of five FS methods that use either filter, wrapper, or embedded approaches. The filter methods are three ranking univariate approaches: chi2, FIS, and MI. The wrapper method uses a selector that benefits from a forward-greedy search engine and a classifier to evaluate the performance of selected subsets. The embedded method, which in principle is a combination of filter and wrapper methods, is based on RFE. The ensemble selector level consists of four approaches based on heterogeneous and homogeneous ensembles through aggregation functions, union and intersection, which benefit from the results of the base-level selectors. Two traditional ML methods, including SVM and KNN, and RF as the ensemble ML method, are employed to evaluate the classification performance of each FS method output.

Our experimental results show that (1) we improved the accuracy of the predictive models by using the proposed FS methods, (2) we identified meaningful and important clinical factors related to prediction of AUD, and (3) we proved that gender disparity must be considered when developing ML techniques and identifying clinical factors related to prediction of AUD. In terms of feature reduction, RFE reduced the number of features from 361 to 131, and this improved the performance of the predictive models. However, it has several disadvantages. Therefore, our proposed UFFS method, which could reduce the number of features to 233 and improve classification performance to an AUROC of 0.98 and an AUPRC of 0.84, has been chosen as the best method. This method used a smaller number of reductions, which enables us to identify more informative clinical and risk factors related to prediction of AUD in medical staff for the prognosis and diagnosis of AUD.

We have identified that diseases related to digestive organs, bones, muscles and connective tissue, and nervous systems, and also the length of stay at the hospital, are highly correlated to the development of a predictive model to identify patients with AUD, though not as much as age and gender. However, age and gender are common features for many diseases (every model is based on these), so the methods used in this paper also identified these as highly correlated with prediction of AUD.

Our experimental results showed that it is important to consider gender disparity while developing predictive models for the identification of patients with AUD. Our results also indicated that there were more than 50% differences among the contributed features in the RF predictive models for each group of gender. This resulted in worsening the predictive accuracy for the Male group.

In summary, the primary contributions of this paper are four-fold: (1) the developed feature selection framework is effective in reducing the features to a more manageable number; (2) some of the developed models demonstrate a high accuracy in prediction AUD as measured by AUROC and AUPRC; (3) the study points to clinical factors that are highly correlated to development of predictive models to identify patients with AUD; and (4) proving that gender disparity should be considered when building predictive models to identify patients with AUD.

## Limitations and future work

One limitation of this study is that the data used to develop the predictive models comes from patients who were admitted to the Gastrointestinal, Neurologic, and Orthopedic departments at OUH. This introduces a risk of bias, which can be observed in the identified clinical factors. An additional limitation of our study is the relatively low numbers of patients and the lack of geographical diversity that results from a single-site study. Since the results of this study are very promising, we plan to address the limitations by conducting a national study with a much larger dataset to improve the predictive accuracy and quality of identified clinical factors related to AUD. Our future study will include data about patients from several Danish regions and hospitals.

Although our framework covers different types of FS methods, it is always relevant to evaluate the performance of other FS methods on EHRs for the prediction of AUD. Recent studies show great predictive performance by deep learning approaches. Although deep learning methods such as deep neural networks act as black boxes and are not suitable for clinical and risk factor discovery, a deep ensemble FS method based on aggregation functions may produce a better result along with clear clinical risk factors for AUD. In such a framework, at least three levels of FS methods will contribute to evaluating the weight of features, and in each level, at least two similar FS methods will be incorporated. This approach will produce several feature sets for the ensemble FS level. In summary, we plan to expand the FS framework in our future national study to include deep learning methods as well.

## Figures and Tables

**Fig. 8:**
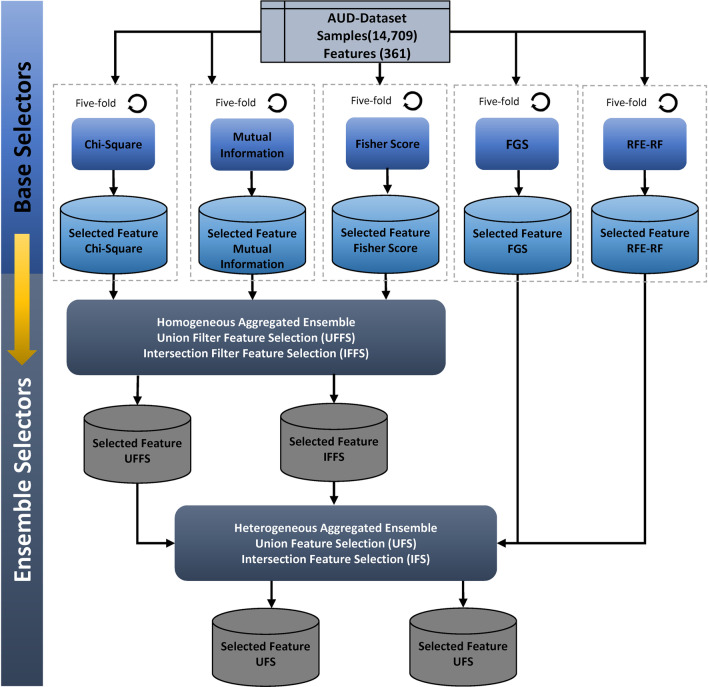
Feature selection workfellow

## Data Availability

The dataset used for this study is not publicly available due to the possibility of compromising individual privacy but is available from the corresponding author on reasonable request.

## Appendix: Feature selection framework

See Fig. 8.

## Footnotes

1 https://open.rsyd.dk.
